# Current Knowledge, Attitudes, and Practice among Health Care Providers in OSCC Awareness: Systematic Review and Meta-Analysis

**DOI:** 10.3390/ijerph18094506

**Published:** 2021-04-23

**Authors:** Noemi Coppola, Michele Davide Mignogna, Immacolata Rivieccio, Andrea Blasi, Maria Eleonora Bizzoca, Roberto Sorrentino, Lorenzo Lo Muzio, Gianrico Spagnuolo, Stefania Leuci

**Affiliations:** 1 Oral Medicine Unit, Department of Neurosciences, Reproductive and Odontostomatological Sciences, University of Naples Federico II, 80131 Naples, Italy; noemi.coppola@unina.it (N.C.); i.rivieccio@outlook.com (I.R.); andreablasi79@gmail.com (A.B.); roberto.sorrentino@unina.it (R.S.); gspagnuo@unina.it (G.S.); stefania.leuci@unina.it (S.L.); 2 Department of Clinical and Experimental Medicine, University of Foggia, 71100 Foggia, Italy; marielebizzoca@gmail.com (M.E.B.); lorenzo.lomuzio@unifg.it (L.L.M.)

**Keywords:** awareness, education, KAP, knowledge, attitude, practice, oral cancer, OSCC, dentists

## Abstract

OSCC remain a global health problem. Lack of awareness leads to inadequate watchfulness regarding early signs/symptoms despite the ease of visual oral inspection. What clinicians know and feel, and how they behave on OSCC is crucial to understand the feasibility and effectiveness of screening programs. The aim of this systematic review was to assess knowledge, attitudes, and practice (KAP) regarding OSCC among health care providers (HCPs). Therefore, a systematic review was conducted with SPIDER and PICO as major tools. A meta-analysis was structured through common items in two comparison groups of medical and dental practitioners. Descriptive statistics and a Mantel–Haenszel test were used to validate data. Sixty-six studies were selected for systematic review, eight of which are useful for meta-analysis. A statistically significant difference was recorded between dentists and medical practitioners for questions regarding: Alcohol (*p* < 0.001); *Elderly* (*p* < 0.012); *Sun exposure* (*p* < 0.0001); *Erythroplakia* (*p* < 0.019); *Red patch* (*p* < 0.010); *White patch* (*p* < 0.020); *Tobacco consultation* (*p* < 0.0001); *Intraoral examination* (*p* < 0.0001) and *Up-to-date knowledge* (*p* < 0.002). Overall, the incidence of OSCC screening is low. Most HCPs feel the need to increase KAP. Data confirmed gaps in KAP, highlighting the need for a more efficient pre- and post-graduation training, necessary to increase competence worldwide.

## 1. Introduction

Oral cancer is a large global health problem where statistical data have changed little over time, with 177,757 deaths out of 377,713 new cases recorded in 2020 and with a low 5-year survival rate of 50% [1,2]. The oral cavity is easily accessible for routine screening through clinical examination; therefore, in theory, dysplastic changes should be straightforwardly detected and diagnosed in the early stages, leading to more effective management. Oral squamous cell carcinoma (OSCC) is the most common type of oral neoplasms, accounting for over 90% of oral cancers. Visual screening for OSCC is quick (requiring only five minutes), simple, inexpensive, and non-invasive, and it causes little discomfort to the patients, whereas the detection of most solid malignancies in their early asymptomatic stages usually requires special, costly, and often invasive techniques [3]. However, most of the oral lesions are detected in their late stage, often too late for any therapeutic treatment [4].

The WHO has listed early screening and prevention as the priority objectives to keep under control OSCC global spread: Early detection, including opportunistic screening of asymptomatic populations and awareness of early signs and symptoms, increases the probability of cure [5]. In this context, health care providers (HCPs) such as dentists, maxillofacial surgeons, general physicians, otolaryngologists (ENTs), and dermatologists play a crucial role [6,7,8] since they are well trained to provide oral examination and to screen the presence of suspicious lesions. This good practice might help not only in primary prevention but also in timely OSCC detection. 

The perception of knowledge, attitudes, and practices (KAP) among HCPs in this field is mainly explored and studied through research projects based on surveys, either face-to-face interviews or questionnaires [9]. The knowledge possessed by a medical community refers to its OSCC understanding; attitude refers to its feelings towards the disease, as well as to any preconceived ideas that it may have towards it; practice refers to the ways in which it demonstrates its knowledge and attitude through actions [10]. Understanding the levels of KAP would allow pre- and post-graduate training programs to be modified according to the needs of the medical community, focusing on the fields where there is a greater need for training [11,12]. 

In our field of interest, the understanding of KAP among HCPs is a key step to minimize OSCC risk, improve prevention and control measures, and apply detection procedures, because oropharyngeal cancers can be recognized at an early stage by visual and tactile examination. On the other hand, the assessment of KAP is also essential because it has a key role in counseling patients regarding OSCC early detection [13].

In this systematic review, all studies measuring OSCC-KAP among dentists and physicians were collected with the purpose to summarize and compare knowledge, feelings, and behaviors among medical (MDs) and dental practitioners (DDSs). 

## 2. Materials and Methods

The systematic review relied on a PRISMA statement with the use of Sample, Phenomenon of Interest, Design, Evaluation, and Research Type (SPIDER) and Population, Intervention, Comparison, Outcome (PICO) tools in order to structure the research question [14,15,16]: “Is there any difference in KAP among HCPs regarding OSCC?”.

### 2.1. Eligibility Criteria

The review included qualitative, quantitative, and mixed-method studies written in the English language. Studies investigating the current knowledge status and/or skills and/or attitudes and/or perceptions and/or practices and/or behaviors of MDs and DDSs were taken into account. 

### 2.2. Search Strategy

The used databases were PubMed and Scopus. The search strategy was based both on medical subject headings (MeSH) and on the following key words, in multiple combinations, which were chosen to reflect the focus of the review: “Oral cancer”, “oral neoplasm”, “oral malignant”, “knowledge”, “awareness”, “early detection”, “prevention”. Studies published up to December 2020 (included), from any year, were sought. In addition, the search was supplemented by searching of the reference lists of the included studies.

### 2.3. Study Selection

Two authors were involved in the literature search. The choice of the reference studies was made firstly on the screening of titles and abstracts of all the articles after the exclusion of duplicates, in an unblinded but independent process. The independent lists were cross-referenced; any disagreement was resolved by consensus or with a third-party reviewer. Then, in line with inclusion and exclusion criteria (Table 1), a full-text eligibility assessment was performed by the two reviewers in a blinded process, after which the process of referencing and citation searching was made. A 100% agreement rate was obtained between the two authors.

### 2.4. Data Extraction and Data Synthesis

A standardized form was used to extract data from the included studies. To assess the aim of the review, the following data were collected: Author’s name, year of publication, purpose of study, sample size, type of HCP’s, and OSCC-related items explored in the questionnaire-based surveys classified in three distinct domains, including knowledge, attitude, practice, and outcomes related to these domains. In particular, the knowledge-related items consisted of eleven statements about risk factors, seven about non-risk factors, six regarding oral potentially malignant disorders (OPMDs), six related to common sites of development, and eleven about clinical presentation. Seventeen statements investigated attitude items. To regard practice items, four statements were associated with physical examination and biopsy, seven with history taking, and one referral to a specialist. A detailed explanation of the explored items in the questionnaires and surveys is reported in Table 2. 

### 2.5. Quality Assessment

The methodological quality of the studies was assessed according to the Strengthening the Reporting of Observational Studies in Epidemiology (STROBE) scale.

Where possible, a meta-analysis was structured through common items in two comparison groups of HCPs.

### 2.6. Statistical Analysis

The assessed items were the following ones: Alcohol, Elderly, Sun exposure, Erythroplakia, Red patch, White patch, Tobacco counseling, Intraoral examination, and Up-to-date knowledge. Overall, for the systematic review purpose, descriptive statistics of selected items among HCPs were obtained, reporting absolute numbers and percentages. In order to evaluate differences in knowledge and perception of each OSCC acknowledged risk factor between DDSs and MDs, meta-analysis was conducted by the Mantel–Haenszel method (random effects model). The possibility of heterogeneity across the studies was assessed with an I^2^ test. The data analysis was performed using a commercially available statistical software (IBM SPSS, IBM Corporation, Armonk, NY, USA) with a *p*-value < 0.05 considered statistically significant.

## 3. Results

**Figure 1:**
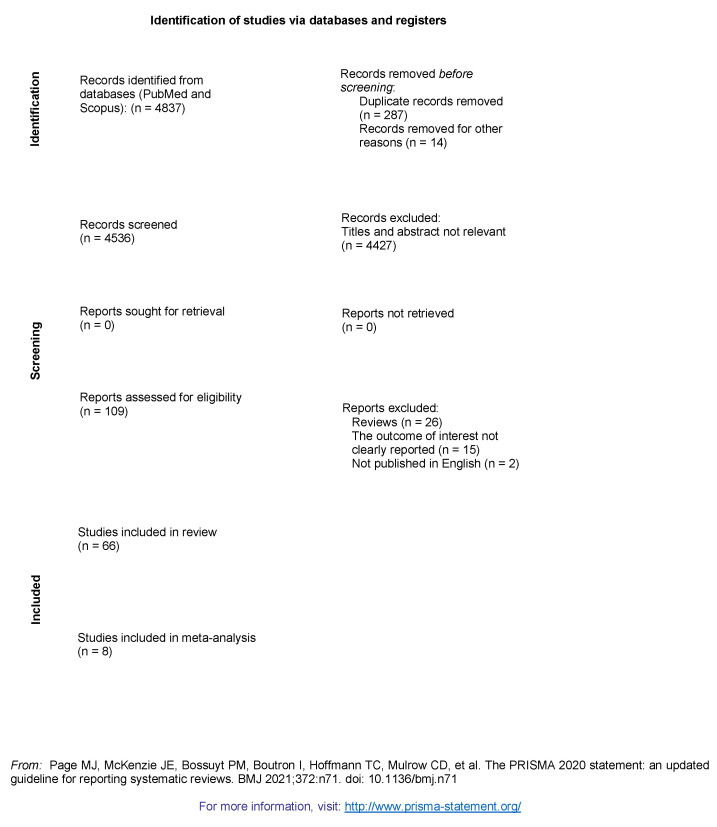
Prisma flow diagram. For more information, visit: http://www.prisma-statement.org/ (accessed on 31 December 2020).

PubMed and Scopus research produced 4837 abstracts. After removing duplicates and, in compliance with the study protocol, a detailed screening of titles and abstracts of the manuscripts was made (Figure 1).

The reporting of literature review on the KAP key question showed 55 studies using SPIDER and 11 using the PICO tool, divided per categories as follows: 50 among dentists [12,17,18,19,20,21,22,23,24,25,26,27,28,29,30,31,32,33,34,35,36,37,38,39,40,41,42,43,44,45,46,47,48,49,50,51,52,53,54,55,56,57,58,59,60,61,62,63,64,65], eight among physicians [66,67,68,69,70,71,72,73], and eight between dentists and physicians [74,75,76,77,78,79,80,81].

Twenty-four studies were conducted in America [17,18,22,39,43,50,51,52,55,56,57,58,59,60,61,62,63,65,67,71,73,77,78,81], 20 in Europe [12,27,38,42,44,45,46,47,48,49,53,54,64,66,68,69,70,72,79,80], 17 in Asia [19,20,23,24,29,30,32,33,34,35,36,37,40,41,74,75,76], two in Africa [21,25], two in Australia [26,31], and one in Japan and Australia [28] (Table 3 and Table 4).

### 3.1. Domain 1—Dental Practitioners

#### 3.1.1. Knowledge

Most studies indicated sound knowledge among DDSs about OSCC risk factors like tobacco (26.3–100%) [18,22,29,40,48,55,60] and past positive OSCC history (75.2–99%) [55,74]. However, considerable variability in knowledge levels was noticed among participants regarding other risk factors, including alcohol (8.1–100%) [22,51], HPV (18.2–93%) [24,28], elderly (21.8–76%) [24,55], diet (6–59.2%) [37,77], and betel quid chewing (25–99.2%) [28,35].

OPMDs correctly identified by DDSs included leukoplakia (28.4–87%) [23,45], erythroplakia (7.7–82%) [23,45], and oral lichen planus (OLP) (5.7–72%) [23,49]. Regarding clinical picture items, DDSs were generally knowledgeable about OSCC being the most common form of oral cancer (52.6–100%) [30,39], positive lymph node characteristics (48.6–86.2%) [29,48], asymptomatic at early stage (21.5–95.6%) [19,48], OSCC diagnosis at III/IV stage (28–94.7%) [43,48], and tongue high-risk area (46.3–96.8%) [19,48]. Considerable variability in knowledge of common sites of development was noticed regarding tongue (20.4–80.9%) [25,76] and floor of mouth (8.2–86%) [32,45].

#### 3.1.2. Practice

Thirty-eight per cent of selected studies assessed history taking [12,19,20,29,33,34,39,40,41,45,47,51,52,53,56,59,62,63,64,65,77,78], asking patients about risk behaviors, family history, and prior OSCC. Regarding physical examination, DDSs identified the following items: Intraoral (51–99.7%) [25,26], extraoral (40.8–83.8%) [25,26], and lymph node (23–80.7%) [26,37] examinations. Most of them usually referred patients to specialists (12–97.2%) [18,41]; some papers described the specialists’ sub-specialty such as oral and maxillofacial surgeons (65% and 77%) [40,53], ENT, themselves, or physicians [18,22,29,51,53,76,79,80].

#### 3.1.3. Attitude

The attitudes were reported in 42 (72%) studies [12,17,18,19,20,22,23,24,25,26,27,28,29,31,33,34,37,38,39,40,41,43,44,45,46,47,48,50,51,52,53,55,57,58,59,61,62,63,65,76,78,81], mainly related to participants’ perception and inclination towards OSCC awareness. DDSs identified the following items: “Visual examination is effective in early detection” (28–98.9%) [29,31], “undergraduate training was adequate” (20.4–78%) [34,62], “up-to-date personal knowledge” (3.6–92.4%) [17,24], “OSCC early diagnosis improves the survival rate” (65.5–99%) [55,81], “key role of dentist” (60.4–98.6%) [18,34], and “need of CE” (31.8–95.8%) [18,38].

### 3.2. Domain 2—Medical Practitioners

#### 3.2.1. Knowledge

Most studies indicated a good level of knowledge among MD about tobacco (78.3–100%) [68,73,76] as an OSCC risk factor. Instead, considerable variability in knowledge levels was noticed among participants regarding other risk factors, including alcohol (34.9–100%) [67,76], HPV (18–82%) [68,77], past positive OSCC (31.5–100%) [68,72], elderly (2.8–87.8%) [69,72], diet (2.3–68.9%) [66,69], sun exposure (3.2–96%) [66,68], and betel quid chewing (0.8–98%) [70,77].

OPMDs correctly identified by MDs included leukoplakia (12.7–91.5%) [70,72], erythroplakia (0.4–62%) [70,71], and OLP (1.2–27.9%) [70,74]. Regarding clinical picture items, physicians were generally knowledgeable about OSCC being the most common form of oral cancer (60.9–93%) [68,72], positive lymph node characteristics (3.2–94%) [66,68], asymptomatic at early stage (27–82%) [68], and ulcers (66.7–100%) [66,67].

Considerable variability in knowledge of common sites of development was noticed regarding tongue (22.8–80.9%) [75,76] and floor of mouth (25.7–75%) [68,76].

#### 3.2.2. Practice

Thirty-seven per cent of studies assessed history taking by MDs [69,70,72,73,77,78]. Regarding physical examination, they identified only intraoral examination (39.4–100%) [68,76]. A minority of MDs preferred to refer to specialists (11.8% and 56%) [70,79], which was usually an ENT consultant (24–59%) [76,80], followed by an oral and maxillo-facial surgeon (18.6% and 42%) [66,70] and an oral medicine specialist (2% and 25.4%) [66,70].

#### 3.2.3. Attitude

The attitude was reported in 9 studies (56%) [66,67,69,70,72,73,76,78,81]. MDs identified the following items: “Visual examination is effective in early detection” (14–61.8%) [66,73], “undergraduate training was adequate” (44% and 53.8%) [67,73], “up-to-date knowledge” (32.6%) [81], “OSCC early diagnosis improves the survival rate” (63.3% and 87.8%) [69,81], “physicians are qualified to perform OSCC examination” (67%) [78], and “need of CE” (84.9% and 99.6%) [68,70].

### 3.3. Meta-Analysis

**Figure 2:**
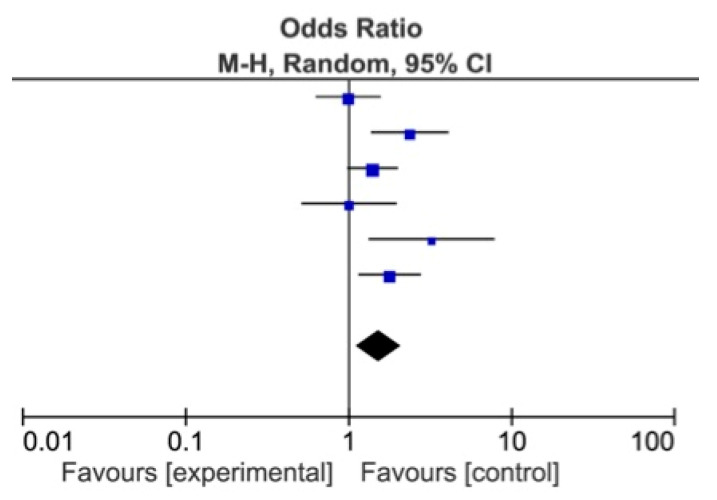
Forest plot for identification of alcohol as oral cancer risk factor among DDs and MDs.

**Figure 3:**
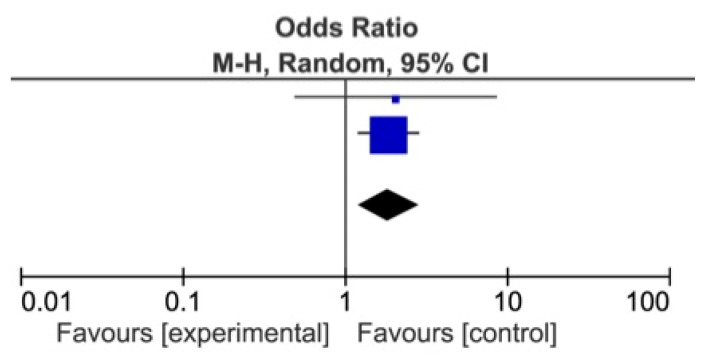
Forest plot for identification of red patches among DDs and MDs.

**Figure 4:**
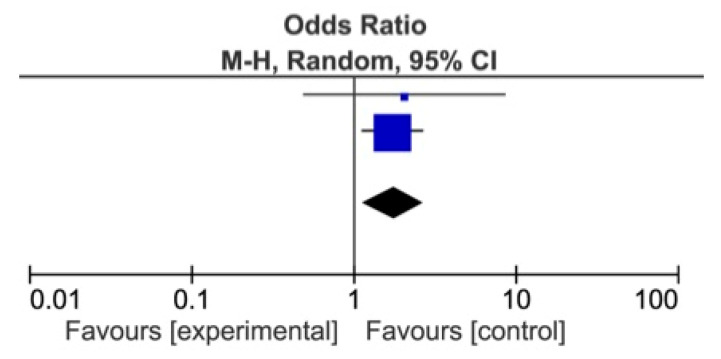
Forest plot for identification of white patches among DDs and MDs.

The meta-analysis showed a statistically significant difference between DDSs and MDs, favoring DDs for identification of *Alcohol* as a risk factor for OSCC (*p* = 0.007), and of Red patches (*p* = 0.004) and *White patches* (*p* = 0.009) as OPMDs (Figure 2, Figure 3 and Figure 4) [74,75,76,77,78,79,80,81].

## 4. Discussion

KAP studies and their related methodologies are one of the best ways to assess health care delivery by identifying gaps in knowledge and facilitating educational processes, with the important advantage of collecting a large amount of qualitative and quantitative data that will be subjected to statistical analysis. While “Knowledge” had more objective items to assess, “Attitude” was difficult to analyze because it was strictly related to acquired characteristics of an individual, including cognitive, affective feeling elements, and a tendency to action [82]. The quality of included studies was varied and heterogeneous and there were dissimilarities in design, samples, and results; data examination from two focus group uncovered several similar themes.

This systematic review revealed that knowledge among DDSs about tobacco and alcohol was satisfactory and highlighted their consolidated role in the etiology of the disease. In addition, among MDs the knowledge on tobacco, alcohol and prior oral cancer was sufficient [66,68,69,70,71,72,73,74,75,76,79,80]. Otherwise, regarding HPV and diet in the DDS group, inadequate level of knowledge was demonstrated by the very small number of articles where they were considered and by the low scores assigned by participants. Furthermore, betel quid chewing and sun exposure, even if recognized by many DDSs, were taken into consideration in a small number of articles, 21% and 36%, respectively. Even in the MD group, the level of knowledge about HPV, elderly, diet, and betel quid chewing was low [66,70,72,77]. Few studies evaluated the “controversial risk factors” [19,35,37,40,46,55,61,75,76] (poor oral hygiene and decay teeth), in line with the current scientific literature, not yet supported by sufficient scientific evidence and therefore not yet a stable part of the clinical diagnostic OSCC management. Regarding knowledge of OPMDs, some particularly surprising data also emerged: Only 50% of the studies among DDSs assessed knowledge of OPMDs [12,18,19,20,22,23,27,30,37,38,39,40,43,44,45,46,48,49,51,55,57,58,60,65,74,75,78,79]; this is a very low value compared to the evidence in the literature. OPMDs was considered as a risk factor by a single article out of the 58 analyzed and recognized as such by 60.9% of DDSs [74]. The data analysis also revealed a significant lack of knowledge regarding oral sub-mucous fibrosis (OSMF) [74], since it was evaluated in one single study; this fact disagrees with the current knowledge which recognizes it as a well-known OPMD [83]. These data are clearly in contrast with current knowledge; in fact, prevention and early detection of such conditions have the potential not only of decreasing the incidence but also of improving the survival of people who develop OSCC [84]. Instead, the majority of studies (75%) among MDs investigated knowledge on OPMDs showing a higher knowledge to recognize leukoplakia [67,68,69,71,72,74,75].

A good level of knowledge among DDSs has also emerged with regard to the common high-risk sites of cancer development such as tongue and floor of the mouth [85]. On the other hand, from the 6 studies analyzing the same item among MDs, a poor expertise to recognize the floor of the mouth as a high-risk area emerged [76]. OSCC knowledge is indispensable for the correct execution of screening program, a pivotal step in early detection. It involves an oral examination with the objective of identifying changes, which may precede or predict, with a high likelihood, the development of the disease [86].

Regarding the clinical picture items, our analysis showed a good level of knowledge among the participants. In particular, a high percentage of DDSs were aware that: (i) The most common form of oral cancer is squamous [19,20,25,30,33,34,41,43,44,46,47,48,55,57,58,60,61,65,74,75,77]; (ii) the patient is asymptomatic in the initial stage [40,43,45,48,55,58,75]; (iii) the tongue is a high-risk area [43,45,48,50,58,60,61], as well as (iv) knowing the characteristics of a positive lymph node [19,20,22,23,40,43,46,48,55,57,60,61,74,75]. Results were positive even among MDs as most physicians identified: (I) Squamous cellular as the most common form of oral cancer [68]; (II) positive lymph node features [68]; (III) OSCC early lesion features [73]; (IV) persistent ulcer as a sign of OSCC [67].

Regarding attitude, reported in 72% and 56% of study among DDSs and MDs respectively, both HCPs identified the same items.

Regarding practice items, a significant gap in knowledge with respect to the diagnostic procedures emerged. DDSs, often despite being aware of the OSCC clinical characteristics, did not perform or were unable to perform proper clinical patients’ management (physical examination, history taking, risk factor examination, and referral). In particular, as part of the physical examination, it was found that DDSs rarely resort to biopsy during their activity, which is crucial in detection. Biopsy technique is an easy-to-learn competence skill, but it is the practical and last result of a previous comprehensive and complex knowledge acquisition in this field; it is important to know how to perform biopsy, but, first of all, why/where/when to perform biopsy. Research shows that few DDSs would perform a biopsy on their patients. However, waiting times for patients to be seen at specialist centers may be long. The diagnostic delay for the patient may have a negative impact on survival and cure rates [87]. Only seven studies among DDSs and two studies among MDs analyzed the habit of referring patients to specialists and there was a higher trend among DDSs than MDs [29,33,40,41,48,50,67,81].

Moreover, data analysis showed that most MDs are interested in history taking [69,72,73,77,78]. To date, the history taking in dentistry field is often an underestimated and neglected aspect. Probably this is due to the fact that during the degree course the efforts are mostly focused on the practical aspects, with consequent loss of the medical background which should be crucial for the clinical algorithm acquisition. Perhaps in Italy after the division of the two-degree programs of “Medicine and Surgery” and “Dentistry and Dental Prostheses”, dental profession has been considered as a separate section of medicine, this way losing the basic knowledge of medicine that should be shared with medical profession. In fact, both professions operate in complete synergy, where systemic diseases can show different signs in the oral cavity and, at the same time, the mouth can be the first site of the onset of systemic diseases. First of all, DDSs are “physicians” of the mouth and nearby structures, also specialized in oral care and they can first detect pathological changes of Head/Neck soft and hard tissues. They must become aware of themselves and of their primary role in patient’s health. In particular, in the academic programs it would be necessary to join the first three-year period of study between dental and medical students, in order to create a common pathway of knowledge and learning. As for the other focus group, also in this case physicians know they play an important role in the OSCC early diagnosis, but they feel they are not updated and lack adequate knowledge.

It was possible to compare KAP of DDSs and MDs for 8 studies used for the meta-analysis. Although the two groups agreed on most of the items, significant differences of opinions were found in 9 out of the 20 items considered (*p* < 0.05 from the Mantel-Haenszel test). In particular, DDSs are better trained to identify the following risk factors—*Alcohol* (*p* < 0.001), *Elderly* (*p* < 0.012) and *Sun exposure* (*p* = 0.0001)—to perform the intraoral examination (*p* < 0.0001), and to recognize the white/red lesions (*p* < 0.020; *p* < 0.010). Instead, MDs are more able to provide tobacco cessation counseling than DDSs. This is due to the fact that smoking is associated with a range of diseases, causing a high level of morbidity and mortality and it is a major preventable cause of death. Quitting smoking has important health benefits and the physicians are in a unique position to help patients quit tobacco [88].

The diversity in methodology and quality of included studies may have compromised the reliability of findings. In fact, the main difficulty encountered has been to standardize data for analysis. The absence of a standardized questionnaire for the evaluation of KAP on OSCC was a barrier for data comparison.

This review identified gaps among HCPs towards OSCC primary and secondary prevention, with a very disappointing scenario. HCPs showed mixed attitudes with inconsistent clinical practices related to routine OSCC screening, patient counseling, and referrals. The referral pattern lacked details, justifying global data on the diagnostic OSCC delay [89]. These findings suggest the need for further education and training on timely diagnosis and referral in association with patient guidance to promote OSCC awareness. Patients’ responsibility in diagnosis delay is only one side of the problem. It is also necessary to analyze the HCPs’ commitment to the OSCC prevention and diagnosis [90].

HCPs play a pivotal role in this setting and it is imperative to improve their knowledge. Actions to be taken could be many and in different areas: (i) Education and awareness campaigns on traditional and emerging risk factors; (ii) implementation of knowledge in undergraduate training for a better understanding of causative factors and pathogenesis; (iii) annually free and mandatory education programs post-graduation. In order to reach as many HCPs as possible, continuing education programs should be a combination of different approaches and media (oral presentation, journals, poster).

As for education, in some studies it emerged that not only the postgraduate training system is insufficient to guarantee adequate preparation, but it is also needed to improve degree programs involving more activities by trainees and greater frequency to oral medicine units, as well as to participate in mandatory national/regional annual thematic meetings [91].

In addition, targeted policies and strategies should be promoted by competent organizations, such as the NHS and National Dental Associations, in order to make people aware of the possibility that nobody is immune to mouth cancer.

## 5. Conclusions

It is mandatory to improve knowledge, attitudes, and practice among DDSs and MDs about OSCC through the actions described above. Only in this way can we hope for a trend inversion, which will lead to an early diagnosis increase, to an improvement in the patient’s survival rate and to a reduction of the negative economic impact on public health systems, in particular due to a large number of cases presented in late stages (III/IV) for the treatment of cancer.

## Figures and Tables

**Table 1:** Inclusion and exclusion criteria.

		Criteria	Inclusion	Exclusion
		Language		Non-English
**S**	**P**	Sample	HCPs involved in OSCC/OPC management	Non-HCPs
**PI**		Phenomenon of interest	OSCC topics	Non-OSCC topics
	**I**	Intervention	Questionnaire-based survey and interview assessing knowledge OR/AND attitude OR/AND practice (See Table 2)	Non-questionnaire-based survey
**D**		Design of study	Cross-sectional studies/comparative cross-sectional studies/RCTs, non-RCTs	Reviews, opinion-based studies, letter to editors, case reports, study protocols
	**C**	Comparison	Comparison of KAP among different HCPs when available	-
**E**	**O**	Evaluation (E) (O)	HCPs’ knowledge status/skills/attitudes/perceptions/views/opinions/practices/behaviors	Unrelated with HCPs’ knowledge status/skills/attitudes/perceptions/views/opinions/practices/behaviors
**R**		Research type (R)	Qualitative studies, quantitative studies, and mixed-method studies	-
		Geographical area of interest	Worldwide	-
		Study focus	Studies investigating the knowledge AND/OR attitudes AND/OR practices/behaviors of HCPs towards oral health topics Studies investigating almost two among knowledge, attitude, and practice Studies investigating the impact of OSCC/OPC educational interventions on HCPs’ knowledge AND/OR attitudes Studies focusing only on data about single categories of HCPs	Studies investigating the OC/OPC knowledge AND/OR attitudes AND/OR practices of medical/dental students Studies investigating the knowledge AND/OR attitudes AND/OR practices of HCPs towards other oral health related topics Studies with inadequate data Studies focusing on aggregated data per individual categories of HCPs

Abbreviations: HCP: Health care practitioner, OSCC: Oral squamous cell carcinoma, KAP: Knowledge, attitude, and practice study, OC: Oral cancer, OPC: Oral and pharyngeal cancer. The bold is necessary to make the terms recognizable.

**Table 2:** OSCC-related items explored in the questionnaire-based surveys.

**Knowledge**
**1. Risk factors**
Tobacco
Alcohol
Prior OSCC
Advanced age
HPV infection
Sun exposure
Diet
Betel quid chewing
Fungal infection
Immunosuppression
Radiotherapy
**2. Non-risk factors**
Family history
Familiar clustering
Ill-fitting prothesis
Hot food and drink
Poor oral hygiene
Use of spicy food
Obesity
**3. OPMDs**
Leukoplakia
Erythroplakia
Lichenoid lesions
Chronic hyperplastic candidiasis
Actinic cheilitis
Oral sub-mucous fibrosis
**4. Common sites of development**
Lip
Tongue
Floor of the mouth
Buccal mucosa
Palate
Gum
**5. Clinical presentation**
OSCC is the most common form of oral cancer
OSCC is asymptomatic at early stage
OSCC is diagnosticated more frequently at advanced stage
Lymph node characteristic of OSCC metastasis
Early OSCC lesions appear as small, painless red area
Ventral lateral border of the tongue most likely to develop OSCC
Submandibular lymph nodes are the first places of metastasis of OSCC
Lung is the most common site of distant metastasis of OSCC
Persistent ulcer, lump, non-healing socket, and/or bleeding gums could be signs of OSCC
Dysphagia
Limited tongue mobility
**Attitude**
Adequate/inadequate OSCC education received at medical/dental school
Quality of OSCC education
Up-to-date knowledge
Need to perform annual OSCC screening examinations for patients > 40 years old
Early detection improves 5-year survival rate
Training level in providing education on smoking cessation
Training level in OSCCC examination/screening
Believe/do not believe dentist/physician/dental hygienist is qualified to perform OSCC examination
Comfortable/uncomfortable during neck lymph nodes palpation
Comfortable/uncomfortable to refer suspicious oral lesions to specialists
Confident/non-confident in diagnosis of OSCC from clinical appearance
Patients’ knowledge level about risk factors
Should/should not inform patients about findings
Comfortable/uncomfortable to perform biopsy
Advise/do not advise patients with suspicious oral lesions
Need of continuous education in the future
Adequate/inadequate preparation to explain the risks of tobacco/alcohol use
**Practice**
Extra/intraoral examination
Lymph nodes palpation
Biopsy
Use of blue toluidine/fluorescent light
Asking about current/previous use of tobacco
Asking about the type and amounts of tobacco products used
Asking about current/previous use of alcohol
Asking about the type and amounts of alcohol use
Asking about personal/family history of cancer
Asking about type of diet
Asking about sun exposure
Refer to a specialist (as oral and maxillofacial surgeons, oral medicine specialists, ENT, physicians, specialized hospital)

Abbreviations: OSCC: Oral squamous cell carcinoma; ENT: Otolaryngologist.

**Table 3:** Published data about dental practitioners’KAP on OSCC.

			Knowledge				Attitude	Practice	
References	Participants	Quality Assessment	Risk Factors	Precancerous Lesions	Clinical Picture	Common Sites of Development	Opinion	History Taking	Physical Examination
Aldossri et al., 2020 [17]	932	>75%	Tobacco 99.5% Alcohol 97.2% Prior OSCC 98.6% HPV 88.2% Elderly 69.6% Diet 31.8%	N.A.	Ulcer 99.7% Red patch 98.6% White patch 98.6% Dysphagia 93.9% Paraesthesia 96% Airway obstruction 89.3% Oral bleeding 86.6% Pain 85.1% Trismus 65.5% Chronic earache 58.1%	N.A.	Visual examination is effective in early detection 97.9% Skills in neck examination 96.1% Up-to-date personal knowledge 92.4% Skills in auxiliary devices 83.2% Skills in RX 76.5% Smoking cessation is effective 39.6% Biopsy is mandatory 35.4% Advice HPV vaccine 29.5% Alcohol cessation is effective 22.3%	N.A.	N.A.
da Silva Leonel et al., 2019 [18]	71	<75%	Tobacco 100% Alcohol 98.6% Sun exposure 97.2% Family history 95.8% Elderly 93% Ill-fitting prothesis 88.7% Emotional stress 78.9% Poor oral hygiene 78.9% Presence of decay teeth 74.6% Diet 59.2% Oral sex 50.7% Hot food and drink 46.5% Drug abuse 21.1%	Leukoplakia 85.9% Candidiasis 5.6% Stomatitis 4.2% Blistering 1.4%	OSCC diagnosis (III/IV stage) 76.1% OSCC 66.2%	Tongue and floor of the mouth 71.9% Buccal mucosa 11.2% Palate 4.2% Gingival 2.8%	Key role of dentist 98.6% Need of CE 95.8% Lack of patients’ knowledge 84.5% Adequate knowledge 66.2% Previous CE courses 49.3% Undergraduate training was adequate 43.7% Diagnostic procedure experience 26.8%	N.A.	Intra- and extraoral 98.6%
Jboor et al., 2019 [19]	177	>75%	Tobacco 97.4% Prior OSCC 94.3% Alcohol 93.2% HPV 85.3% Betel quid chewing 74.5% Elderly 72.8% Sun exposure 63.3% Gutka use 49.1% Diet 28.2%	Erythroplakia & Leukoplakia 53.7%	OSCC 84.2% Positive lymph node 75.7% Red patch 66.7% Tongue high-risk area 46.3% OSCC diagnosis (III/IV stage) 39% OSCC time diagnosis > 60 years 31.6% Asymptomatic at early stage 21.5%	Tongue 77.9% Floor of the mouth 54.2%	Smoking cessation is effective 90% Up-to-date personal knowledge 66.7% Visual examination is effective in early detection 48.6%	Tobacco 92.6% Prior OSCC 86.8% Family history 80.6% Tobacco products 80% Alcohol products 46.8% Alcohol 66.3%	N.A.
Nazar et al., 2019 [20]	289	>75%	Tobacco 99.7% Alcohol 99.7%	Erythroplakia and Leukoplakia 97.9%	OSCC 80.6% Positive lymph node 74.3% Asymptomatic at early stage 31.3%	Tongue and Floor of the mouth 80.3%	Need of CE 92.4% Up-to-date personal knowledge 55% Visual examination is effective in early detection 38%	Tobacco 62% Alcohol 17%	Intra- and extraoral 50%
Khattab et al., 2018 [21]	400	<75%	N.A.	N.A.	N.A.	N.A.	N.A.	N.A.	Intra- and extraoral 37.5% Lymph nodes 26.5% Biopsy 27.5%
Pavão Spaulonci et al., 2018 [22]	Senior GDPs 105; junior GDPs 84	>75%	Junior: Tobacco 100%/Alcohol 96.4%/Family history 95.2%/Sun exposure 90.5%/HPV 84.5% Other malignancies 83.3%/Ill-fitting prothesis 60.7%/Emotional stress 56%/Oral sex 51.2%/Presence of decay teeth 44%/Hot food and drink 40.5%/Poor oral hygiene 40.5%/Drug abuse 34.5%/Diet 31%/Spicy food 23.8%/Obesity 16.7% Senior: Tobacco 100%/Alcohol 100%/Family history 95.2%/Ill-fitting prothesis 93.3%/HPV 92.4%/Sun exposure 81.9% Other malignancies 79%/Presence of decay teeth 76.2%/Hot food and drink 74.3%/Emotional stress 67.6%/Poor oral hygiene 64.8%/Oral sex 59%/Diet 47.6%/Spicy food 34.3%/Drug abuse 28.6%/Obesity 16.2%	Junior: Leukoplakia 73.8% Senior: Leukoplakia 75.2%	Junior: Ulcer 85.7% Positive lymph node 69% OSCC 67.9% Junior: Ulcer 85.7% Positive lymph node 69.5% OSCC 64.8%	Junior Tongue 59.5% Senior Tongue 50.5%	Junior Previous CE courses 48.8% Undergraduate training was adequate 70.2% Up-to-date personal knowledge 54.8% Junior Previous CE courses 31.4% Undergraduate training was adequate 43.8% Up-to-date personal knowledge 51.4%	N.A.	Junior Intra- and extraoral examination at 1st visit 78.6% Senior Intra- and extraoral examination at 1st visit 85.7%
Hashim et al., 2018 [23]	298	>75%	Tobacco 99% Prior OSCC 92.3% Alcohol 87.30% HPV 76.6% Sun exposure 73.2% Elderly 60.9% Diet 43.8%	Leukoplakia 28.4% Candidiasis 19.1% Actinic cheilitis 18.6% Erythroplakia 7.7% OLP 5.7%	Ulcer 87.6% Positive lymph node 82.9% White patch 79.9% Dysphagia and limited tongue mobility 68.9% Lump 66.9% Red patch 63.2% Non-healing socket 35.1%	Tongue 30.1% Floor of the mouth 18.7% Palate 7%	Need of CE 84.9% Previous CE courses 48% Visual examination is effective in early detection 31.14% Biopsy is mandatory 9.9%	N.A.	Scalpel biopsy 40% Brush biopsy 20.4% Toluidine blue 6% Fluorescent imaging 5.7% Exfoliative cytology 5%
Kogi et al., 2018 [24]	110		Other malignancies 52.7% Tobacco 41.8% Elderly 21.8% HPV 18.2% Alcohol 13.6% Diet 10.9%	N.A.	N.A.	N.A.	Need of CE 86.4% Up-to-date personal knowledge 3.6%	N.A.	Intra- and extraoral examination at 1st visit 43.6%; at recall 32.7%
Ahmed et al., 2017 [25]	130	>75%	Family history 64.6% Comorbidities 60% HPV 60% Elderly 58.4% Ill-fitting prothesis 41.6% Tobacco 38.9% Alcohol 38.9% Diet 29.2%	N.A.	OSCC 90% Ulcer 83.2% Red patch 80.5% White patch 80.5% Swelling 43.4% Pain 13%	Lips 22.1% Tongue 20.4% Floor of the mouth 19.5%	Need of CE 95.6% Need of referral guidelines 88.5% Visual examination is effective in early detection 46% Adequate knowledge 64.7% Undergraduate training was adequate 27.4% Up-to-date personal knowledge 26.7%	N.A.	Lymph nodes 57% Intraoral 51% Extraoral 40.8% Biopsy 26.5% RX 20.4%
Mariño et al., 2017 [26]	241	>75%	Tobacco 99.4% Betel quid chewing 98.2% Prior OSCC 97% Alcohol 94.6%	N.A.	N.A.	N.A.	Visual examination is effective in early detection 95.2%	N.A.	Intraoral 99.7% Oropharynx 92.3% Extraoral 83.8% Lymph nodes 80.7%
Kebabcıoğlu et al., 2017 [27]	170	>75%	Tobacco 98.8% Prior OSCC 95.3% Alcohol 91.2% HPV 90% Sun exposure 86.5% Betel quid chewing 80.6% Elderly 56.5% Diet 52.4%	Erythroplakia and leukoplakia 64.1%	OSCC 64.7% White patch 35.9% Red patch 26.5% OSCC time diagnosis >60 yrs 12.9%	Tongue and floor of the mouth 37.1% Palate and floor of mouth 11.2% Palate 2.4%	Lack of patients’ knowledge 85.9% Skills in neck examination 70% Visual examination is effective in early detection 53%	N.A.	N.A.
Haresaku et al., 2016 [28]	Japanese 137; Australian 259	>75%	Japanese: Tobacco 90% Family history 74% Alcohol 52% HPV 38% Betel quid chewing 25% Caffeine 15% Australian: Betel quid chewing 98% Tobacco 98% Alcohol 94% HPV 93% Family history 75% Caffeine 5%	N.A.	N.A.	N.A.	Japanese Need of CE > 90% Visual examination is effective in early detection 76.8 %	N.A.	Japanese Intraoral 89.3% Extraoral 35.7% Lymph nodes 10.7% Oropharynx 10.7% Australian Intraoral 98.4% Extraoral 80.5% Lymph nodes 50.8% Oropharynx 23.8%
Navabi et al., 2016 [29]	313	>75%	Alcohol 26.3% Tobacco 26.3%	N.A.	OSCC 66.7% OSCC time diagnosis >60 yrs 66.7% Positive lymph node 48.6%	Tongue and lips 26.7%	Ease for referral 92.7% Undergraduate training was adequate 41.3% Visual examination is effective in early detection 28% Smoking cessation is effective 27.9% Up-to-date personal knowledge 26.7% Dentist skills in visual inspection 20.2% Physician skills in visual inspection 13.4%	Tobacco 87.2% Tobacco products 76.5% Alcohol 76.5% Prior OSCC 75.9% Family history 75.2%	N.A.
Akbari et al., 2015 [30]	GDPs 55; specialists 18	<75%	GDPs Tobacco 92.7% Specialists Tobacco 88.9%	GDPs Leukoplakia 58.2% Specialists Leukoplakia 66.7%	GDPs Submandibular lymph nodes as first place of metastasis 89.1%/OSCC 87.2%/Lung as first place of distant metastasis 67.3%/Two weeks is minimum time to differentiate cancer from inflammation 63.6%/Lower lip is related with better prognosis of oral cancer 49.1%/Minor salivary tumor commonly placed in lateral posterior palate 47.3% Specialists Submandibular lymph nodes as first place of metastasis 100%/OSCC 100%/Lung as first place of distant metastasis 88.9%/Two weeks is minimum time to differentiate cancer from inflammation 94.4%/Lower lip is related with better prognosis of oral cancer 77.8%/Minor salivary tumor common placed in lateral posterior palate 61.1%	GDPs Tongue 70.9% Floor of the mouth 56.4% Specialists Tongue 88.9% Floor of the mouth 88.9%	N.A.	N.A.	N.A.
Hassona et al., 2015 [74]	165	>75%	Tobacco 97.6% Prior OSCC 75.2% Alcohol 64.2% OPMDs 60.6% Betel quid chewing 53.9% Comorbidities 43% HPV 36.4 % Sun exposure 30.3% Elderly 26.7% Diet 21.8%	Leukoplakia 71.5% Erythroplakia 53.3% Candidiasis 42.4% OSMF 33.3% OLP 28.5% Actinic cheilitis 21.8%	OSCC time diagnosis >60 yrs 81.8% Positive lymph node 71.5% Ulcer 68.5% White patches 62.4% Dysphagia and limited tongue mobility 60.6% Red patch 59.4% Lump 58.8% Non-healing socket 38.2%	N.A.	N.A.	N.A.	Scalpel biopsy 84.8% Fluorescent imaging 68.5% Exfoliative cytology 46.7% Brush biopsy 28.5% Toluidine biopsy 24.2%
Allen et al., 2015 [31]	640	<75%	N.A.	N.A.	N.A.	N.A.	Visual examination is effective in early detection 98.9% Key role of dentist 90.9% Key role of dental hygienist 69% Key role of physician 48.2%	N.A.	Intra- and extraoral examination at 1st visit 94.5%; at recall 85.7%
Anandani et al., 2015 [32]	83	>75%	Betel quid chewing 34.1% Tobacco 27.7% Alcohol 14.1% Chronic disease 14.3% Family history 6.1%	N.A.	N.A.	Labial mucosa 26.2% Tongue 19.7% Floor of the mouth 8.2% Palate 6.6%	N.A.	N.A.	N.A.
Alaizari et al., 2014 [33]	800	>75%	Tobacco 96.4% Shammah usage 91.9% Betel chewing 79.2% Prior OSCC 76.9% Alcohol 73.3% Ill-fitting prothesis 70.1% Comorbidities 65.2% HPV 66.1% Elderly 48% Diet 41.6% Sun exposure 67% Obesity 24%	N.A.	OSCC 82.81%	Tongue and floor of the mouth 45.7%	Ease for referral 94.1% OSCC early diagnosis improves the survival rate 87.3% Need of CE 86% Biopsy is mandatory 75.1% Visual examination is effective in early detection 72.4% Smoking cessation is effective 72% Skills in neck examination 68.3% Up-to-date personal knowledge 47.1%	Tobacco 79.6%	Biopsy 75.1% Intra- and extraoral 68.3% Lymph nodes 68.3%
Mehdizadeh et al., 2014 [34]	124	>75%	N.A.	N.A.	OSCC 81.2%	Tongue 59.6% Floor of the mouth 58.8%	Need of CE 94% Delay in OSCC diagnosis 74.8% Key role of dentist 60.4% ENT has a key role in OSCC diagnosis 37.2% ENT has a key role in OSCC treatment 27.4% Undergraduate training was adequate 20.4%	Clinical chart 90.4% Recommendations in elderly 70% Addiction 67.6% Family history 52% Biopsy 37%	Intra- and extraoral 84.8% Lymph nodes 74.2%
Saleh et al., 2014 [35]	362	<75%	Tobacco 99.4% Betel quid chewing 99.2% Alcohol 88.9% HPV 67.2%	N.A.	Ulcer 97% White patch 93.1% Red patch 93.1% Gingival bleeding 67.1%	N.A.	N.A.	N.A.	Intra- and extraoral 84.8%
Ramaswamy et al., 2014 [36]	450	>75%	Tobacco 94%	N.A.	Ulcer 50% Red patch 50%	N.A.	N.A.	N.A.	Intra- and extraoral 90%
Razavi et al., 2013 [37]	139	>75%	Tobacco 97% Alcohol 78% Sun exposure 72% Iron deficiency 62% Ill-fitting prothesis 22% Poor oral hygiene 17% Diet 6%	Erythroplakia and leukoplakia 50%	Positive lymph node 67% Asymptomatic at early stage 45% Red patch 33% OSCC time diagnosis >60 yrs 33%	Tongue and floor of the mouth 51%	Key role of dentist 71% Undergraduate training was adequate 36% Adequate post graduate training 16%	N.A.	Intra- and extraoral 11% Lymph nodes 23% Biopsy 6%
Pentenero et al., 2013 [38]	450	>75%	Prior OSCC 51.1%	Leukoplakia 79.6% Erythroplakia 57.1%	N.A.	Tongue 74.4% Floor of the mouth 72.9% Palate 18.7%	Up-to-date personal knowledge 83.1% Need of CE 31.8%	N.A.	Intraoral 84%
Rocha–Boulevas et al., 2012 [39]	93	<75%	Prior OSCC 78.4% Tobacco 78.4% Alcohol 56.9% Elderly 44.1% Diet 17.2%	Erythroplakia and leukoplakia 51.61%	OSCC 52.6% Red patch 51.6% OSCC time diagnosis >60 yrs 47.3%	Tongue and floor of the mouth 18.2%	Smoking cessation is effective 74.19% Alcohol cessation is effective 67.74% Skills in neck examination 66.67% Lack of patients’ knowledge 47.3% Visual examination is effective in early detection 39.78%	Alcohol products 88.1% Alcohol 82.2% Prior OSCC 59.14% Tobacco products 50.54% Tobacco 49.4% Family history 22.58%	N.A.
Joseph et al., 2012 [40]	153	>75%	Tobacco 100% Prior OSCC 97.3% Betel quid chewing 89% Alcohol 88.8% Sun exposure 82.7% HPV 71.2% Elderly 60.3% Diet 52.7%	Erythroplakia and leukoplakia 93.2%	Asymptomatic at early stage 90.7% OSCC diagnosis (III/IV stage) 75% Positive lymph node 70.1% Visual inspection is the most effective screening method 50.4%	Tongue and floor of the mouth 85%	OSCCC early diagnosis improves survival rate 98% Ease for referral 89.5% Skills in neck examination 72.5% Up-to-date personal knowledge 51.6% Smoking cessation is effective 38.6% Visual examination is effective in early detection 38.6% Previous CE courses 30.1% Alcohol cessation is effective 20.3%	Tobacco 65% Alcohol 21.6%	Intraoral 86.3% Biopsy 62.9%
Vijay Kumar et al., 2012 [41]	240	<75%	Alcohol 99% Tobacco 78.3% Ill-fitting prothesis 53.7% Sun exposure 45% Elderly 31.2%	Blistering 3%	OSCC 96% White patch 82% OSCC time diagnosis >60 yrs 59% Red erosion 9%	Buccal mucosa 83%	Ease for referral 98.7% Annual visual inspection for patients over 40 is mandatory 67.9% Visual examination is effective in early detection 68.3% Skills in neck examination 50.4% Up-to-date personal knowledge 39.1%	Tobacco 68% Alcohol 68%	Intra- and extraoral 37% Lymph nodes 37% Biopsy 24%
Seoane et al., 2012 [42]	791	>75%	N.A.	N.A.	N.A.	N.A.	N.A.	N.A.	Intraoral 90.3% Biopsy 28.7%
Maybury et al., 2012 [43]	463	>75%	Tobacco 98% Prior OSCC 97% Alcohol 95% HPV 88% Elderly 71% Sun exposure 64% Diet 35%	Erythroplakia and leukoplakia 42%	OSCC 83% Asymptomatic at early stage 80% Red patch 81% Positive lymph node 77% Tongue high-risk area 72% OSCC time diagnosis >60 yrs 30% OSCC diagnosis (III/IV stage) 28%	Tongue 59%	Up-to-date personal knowledge 81% Visual examination is effective in early detection 94% Skills in neck examination 79% Smoking cessation is effective 32% Alcohol cessation is effective 15%	N.A.	N.A.
Alami et al., 2012 [75]	55	>75%	Tobacco 92% Alcohol 83% Sun exposure 67% Elderly 62% HPV 47% Diet 33%	Erythroplakia 47.3% Leukoplakia 40% OLP 14.5% Nicotinic stomatitis 14.5%	OSCC 98.2% Red or white patch 94.5% Asymptomatic at early stage 90.9% Positive lymph node 80% OSCC diagnosis (III/IV stage) 75.9%	Floor of the mouth 45.5% Tongue 29.1% Lips 29.1%	N.A.	N.A.	N.A.
Borhan–Mojabi et al., 2011 [76]	86	>75%	Tobacco 78.3% Alcohol 34.9%	N.A.	N.A.	Tongue 80.9% Lips 28.3% Floor of the mouth 25.7%	Lack of patients’ knowledge 40.7%	N.A.	Intraoral 79.15%
Hertrampf et al., 2011 [44]	306	>75%	Tobacco 99% Prior OSCC 95.1% Alcohol 92.8% Sun exposure 68% Elderly 60.5% HPV 57.8% Diet 19%	Erythroplakia and leukoplakia 67.6%	OSCC 86.9% Need of three negative follow-ups 84.6% OSCC diagnosis (III/IV stage) 81% Tongue high-risk area 67.6% Red patch 59.5% OSCC time diagnosis >60 yrs 47.7% Asymptomatic in the early stages 47.1%	Floor of the mouth 76.1% Tongue 70.3%	OSCC early diagnosis improves the survival rate 98.4%	N.A.	Intraoral 84.3%
Decuseara et al., 2011 [45]	254	>75%	Alcohol 98% Tobacco 98% Prior OSCC 83% Sun exposure 79% HPV 60% Elderly 53% Radiotherapy 39% Spicy food 33% Diet 28% Hot food and drink 9%	Leukoplakia 87% Erythroplakia 82% Erythroplakia and leukoplakia 80%	Asymptomatic at early stage 95% Tongue high-risk area 87% OSCC diagnosis (III/IV stage) 86% OSCC time diagnosis >60 yrs 42%	Floor of the mouth 86% Tongue 70%	OSCC early diagnosis improves the survival rate 95% Visual examination is effective in early detection 73% Skills in neck examination 55% Smoking cessation is effective 33% Alcohol cessation is effective 13%	Tobacco 75% Prior OSCC 70% Alcohol 45%	Intra- and extraoral 97%
Hertrampf et al., 2010 [46]	306	>75%	Tobacco 99% Alcohol 93% Prior OSCC 95% Sun exposure 68% Elderly 61% HPV infection 58% Diet 19%	Erythroplakia and leukoplakia 67%	OSCC 87% Need of three negative follow-ups 85% OSCC diagnosis (III/IV stage) 81% Positive lymph node 71% Tongue high-risk area 68% Red patch 60% Asymptomatic at early stage 47% OSCC time diagnosis >60 yrs 46%	Floor of the mouth 76% Tongue 70%	OSCC early diagnosis improves the survival rate 90%	N.A.	Intraoral 70%
Reed et al., 2010 [77]	288	<75%	Tobacco 90% Betel quid chewing 88% Alcohol 45% HPV 26% Diet 6%	N.A.	N.A.	N.A.	N.A.	Tobacco 70%	Intraoral 81%
Klosa et al., 2010 [47]	306	>75%	N.A.	N.A.	N.A.	N.A.	Annual visual inspection for patients over 40 is mandatory 84% Dentists are qualified to perform OSCC examination 71% Visual examination is effective in early detection 63%	Tobacco 65% Prior OSCC 65% Tobacco products 45 Family history 40% Alcohol 35% Alcohol products 25%	Intra- and extraoral 28%
López–Jornet et al., 2010 [48]	340	>75%	Tobacco 100% Alcohol 96.4% Prior OSCC 95.5% Ill-fitting prothesis 95.5% Family history 87.1% Poor oral hygiene 77.6% Elderly 69.4% Diet 52.6% Spicy foods 40.8% Obesity 14.4%	Erythroplakia and leukoplakia 95%	Tongue high-risk area 96.8% Asymptomatic at early stage 95.6% OSCC diagnosis (III/IV stage) 94.7% OSCC 90.6% Positive lymph node 86.2% Red/white patch 89.7% OSCC time diagnosis >60 yrs 72.6%	Tongue and floor of the mouth 89.1%	Dentists are qualified to perform OSCC examination 94.7% Ease for referral 90.9% Annual visual inspection for patients over 40 is mandatory 89.7% Skills in neck examination 52.6% Up-to-date personal knowledge 49.7% Physicians are qualified to perform OC examination 41.8% Smoking cessation is effective 41.5% Alcohol cessation is effective 27.6% Dental hygienists are qualified to perform OC examination 13.8%	N.A.	N.A.
Seoane–Leston et al., 2010 [49]	440	>75%	Diet 18.6%	Leukoplakia 78% OLP 72%	N.A.	N.A.	N.A.	N.A.	N.A.
Applebaum et al., 2009 [78]	274	>75%	N.A.	Erythroplakia and leukoplakia 34%	N.A.	N.A.	Dentists are qualified to perform OSCC examination 96% Visual examination is effective in early detection 85% Lack of patients’ knowledge 79.5% Skills in neck examination 66.67% Up-to-date personal knowledge 50% Physicians are qualified to perform OC examination 45% Smoking cessation is effective 24% Alcohol cessation is effective 12%	Tobacco 85.5% Prior OSCC 85% Family history 56% Alcohol 51% Tobacco products 34% Alcohol products 34%	N.A.
Mahalaha et al., 2009 [50]	34	>75%	N.A.	N.A.	OSCC 79.4% Red patch 76.5% Tongue high-risk area 70% Asymptomatic at early stage 67.6% Positive lymph node 56.3% OSCC time diagnosis >60 yrs 30.3% Persistent ulcer 26.5% Need of three negative follow-ups 6.2% Bleeding area 2.9% Pain 2.9% Swelling 2.9%	Tongue and floor of the mouth 62%	Annual visual inspection for patients over 40 is mandatory 100% Ease for referral 96.9% Dentists are qualified to perform OSCC examination 96.9% Visual examination is effective in early detection 84.4% Skills in neck examination 71.9% Up-to-date personal knowledge 70% Undergraduate training was adequate 65.7%	N.A.	Intra- and extraoral examination at 1st visit 83% at recall 73% Lymph nodes 57%
Colella et al., 2007 [12]	457	>75%	Tobacco 94.1% Prior OSCC 89.5% Alcohol 79.2% Elderly 47.9% Diet 25.8%	Erythroplakia and leukoplakia 53.8%	N.A.	Tongue and floor of the mouth 32%	Smoking cessation is effective 80.9% Alcohol cessation is effective 76.5% Visual examination 53.8% Skills in neck examination 66.8% Lack of patients’ knowledge 39.6%	Tobacco 81.8% Prior OSCC 78.6% Alcohol 71.9% Alcohol products 59.9% Tobacco products 55.6% Family history 47.9%	Intra- and extraoral examination at 1st visit 52.3%
LeHew et al., 2007 [51]	518	>75%	Elderly 47.9% Family history 31.3% Alcohol 8.1% Tobacco 1.2%	Leukoplakia 83.6% Erythroplakia 72%	OSCC 74.7% Positive lymph node 64.3% White patch 53.9% OSCC time diagnosis >60 yrs 20.1% Red patch 31.5%	Tongue 77.4% Floor of the mouth 72% Buccal mucosa 26.3%	Need of CE 74.5%	Tobacco 76.1% Tobacco products 63.5% Alcohol 47.4% Alcohol products 28.8%	Intra- and extraoral 92.3% Lymph nodes 71.5%
Gajendra et al., 2006 [52]	499	>75%	Tobacco 90% Alcohol 80% Sun exposure 60% Elderly 55% Betel quid chewing 52% Diet 25% Gutka consumption 16%	N.A.	OSCC time diagnosis >60 yrs 33%	N.A.	Visual examination is effective in early detection 82% Previous CE courses 80% Skills in neck examination 75% Up-to-date personal knowledge 72% Lack of patient’s knowledge 65% Dentist skills in visual inspection 53% Dental hygienist skills in visual inspection 38% Smoking cessation is effective 20% Alcohol cessation is effective 15%	Prior OSCC 79% Tobacco 70% Tobacco products 58% Family history 57% Alcohol 45% Alcohol products 31%	Intra- and extraoral 85%
Kujan et al., 2006 [53]	143	<75%	N.A.	N.A.	N.A.	N.A.	Undergraduate training was adequate 51%	Prior OSCC 74.2% Tobacco 67.1% Tobacco products 55.2% Alcohol 41.3% Betel quid chewing 39.8% Alcohol products 32.8% Family history 21% Diet 25.3% Sun exposure 10.5%	Intra- and extraoral 92%
Seoane et al., 2006 [54]	32	<75%	N.A.	N.A.	N.A.	N.A.	N.A.	N.A.	Scalpel biopsy 96.9% Intraoral examination 87.5% Toluidine blue 9.4%
Patton et al., 2005 [55]	584	>75%	Tobacco 100% Prior OSCC 99% Alcohol 95% Elderly 76% Sun exposure 74% HPV 60% Diet 39%	Erythroplakia and leukoplakia 73%	Need of three negative follow-ups 98% OSCC 84% Red patch 82% Asymptomatic at early stage 79% Positive lymph node 70% Tongue high-risk area 77% OSCC diagnosis (III/IV stage) 53% OSCC time diagnosis >60 yrs 29%	Floor of the mouth 79% Tongue 78%	OSCC early diagnosis improves survival rate 99%	N.A.	Intraoral 83%
Cruz et al., 2005 [56]	904	<75%	N.A.	N.A.	N.A.	N.A.	N.A.	Tobacco 77% Tobacco products 66% Alcohol 54.5% Alcohol products 36%	Intra- and extraoral examination at 1st visit 86%; at recall 80%
Alonge et al., 2004 [57]	158	>75%	Tobacco 98% Alcohol 98% Prior OSCC 93% Hot food and drink 75% Elderly 74% Spicy food 73% Family history of cancer 70% Obesity 63% Poor oral hygiene 42% Diet 37% Ill-fitting prothesis 33%	Erythroplakia and leukoplakia 31%	OSCC 84% Positive lymph node 79% Red patch 76% Asymptomatic at early stage 76% Tongue high-risk area 68% Lip cancer related to the sun 76% OSCC diagnosis (III/IV stage) 53% OSCC time diagnosis >60 yrs 39%	Tongue and floor of the mouth 51%	Need of CE 81% Undergraduate training was adequate 75% Previous CE courses 64%	N.A.	Intra- and extraoral examination at 1st visit 67% Recall visit 54% Lymph nodes 36%
Macpherson et al., 2003 [79]	225	>75%	Tobacco 94% Alcohol 90% HPV 35% Fungal infections 33%	Leukoplakia 79% Erythroplakia 67%	N.A.	N.A.	N.A.	N.A.	N.A.
Clovis et al., 2002 [58]	British Columbia 401 Nova Scotia 269	<75%	Tobacco 99.4% Prior OSCC 96.6% Alcohol 90.4% Elderly 78.7% Sun exposure 70.1% HPV 53.1% Diet 34%	Erythroplakia and leukoplakia 76%	Need of three negative follow-ups 92.4% OSCC 83.4% Asymptomatic at early stage 78.4% Red patch 77.3% Tongue high-risk area 75.7% Positive lymph node 68.1% OSCC diagnosis (III/IV stage) 54.4% OSCC time diagnosis >60 yrs 45.7%	Tongue 78.7% Floor of the mouth 66.6%	OSCC early diagnosis improves the survival rate 97.8% Up-to-date personal knowledge 56.8%	N.A.	Intraoral 80.6%
Clovis et al., 2002 [59]	British Columbia 401; Nova Scotia 269	<75%	N.A.	N.A.	N.A.	N.A.	British Columbia Visual examination is effective in early detection 83.1% Skills in neck examination 74.8% Undergraduate training was adequate 68.5% Smoking cessation is effective 11.4% Alcohol cessation is effective 5.3% Nova Scotia Visual examination is effective in early detection 79.5% Skills in neck examination 69.4% Undergraduate training was adequate 68.3% Smoking cessation is effective 7.8% Alcohol cessation is effective 5.3%	British Columbia Prior OSCC 93.1% Tobacco 76.8% Family history 69.6% Tobacco products 62.4% Alcohol 35.65 Alcohol products 17.7% Nova Scotia Tobacco 82% Prior OSCC 87.4% Family history 64.5% Tobacco products 60.1% Alcohol 39.3% Alcohol products 23.4%	British Columbia Intra- and extraoral examination at 1st visit 71.2%; at recall 54.5% Lymph nodes 27.4% Nova Scotia Intra- and extraoral examination at 1st visit 69.9%; at recall 45.7% Lymph nodes 26.2%
Canto et al., 2001 [60]	508	>75%	Tobacco 100% Prior OSCC 97% Alcohol 95% Elderly 68% Sun exposure 62% Diet 30%	Erythroplakia and leukoplakia 32%	OSCC 82% Red patch 81% Asymptomatic at early stage 76% Positive lymph node 76% Tongue high-risk area 71% OSCC diagnosis (III/IV stage) 50% OSCC time diagnosis >60 yrs 35%	Tongue and floor of the mouth 62%	N.A.	N.A.	N.A.
Greenwood et al., 2001 [80]	143	>75%	Tobacco 90.7% Betel quid chewing 60.8% Alcohol 45.7%	N.A.	N.A.	N.A.	N.A.	N.A.	Intra- and extraoral 68.2%
Yellowitz et al., 2000 [61]	3200	>75%	Tobacco 99.7% Alcohol 92.7% Prior OSCC 96.4% Elderly 70% Sun exposure 64% Diet 33%	Erythroplakia and leukoplakia 37%	OSCC 83% Red patch 80% Asymptomatic at early stage 76% Tongue high-risk area 71% Positive lymph node 69% OC diagnosis (III/IV stage) 51% OSCC time diagnosis >60 yrs 33%	Tongue and floor of the mouth 54%	Need of CE 84% Up-to-date personal knowledge 68%	N.A.	Intraoral 81%
Horowitz et al., 2000 [62]	3200	>75%	N.A.	N.A.	N.A.	N.A.	Visual examination is effective in early detection 88% Undergraduate training was adequate 78% Skills in neck examination 72% Smoking cessation is effective 28% Alcohol cessation is effective 11%	Prior OSCC 91% Tobacco 83.5% Tobacco products 72% Family history 65% Alcohol 55% Alcohol products 33%	Intra- and extraoral examination at 1st visit 81% at recall 68% Lymph nodes 30%
Horowitz et al., 2000 [63]	243	>75%	N.A.	N.A.	N.A.	N.A.	Visual examination is effective in early detection 92.6% Skills in neck examination 76.5% Undergraduate training was adequate 74% Smoking cessation is effective 25% Alcohol cessation is effective 11.5%	Prior OSCC 92.1% Tobacco 84.2% Tobacco products 70.2% Family history 69.2% Alcohol 60.9% Alcohol products 35.8%	Intra- and extraoral examination at 1st visit 83.7% at recall 78.3% Lymph nodes 34.3%
Warnakulasuriya et al., 1999 [64]	2519	<75%	N.A.	N.A.	N.A.	N.A.	N.A.	Tobacco 50.2% Alcohol 19.3%	Intra- and extraoral 84% Biopsy 21%
Yellowitz et al., 1998 [65]	243	<75%	Tobacco 99.6% Sun exposure 97.9% Prior OSCC 95.7% Alcohol 90.8% Elderly 68.9% Ill-fitting prothesis 64.6% Poor oral hygiene 47.2% Diet 33.6%	Erythroplakia and leukoplakia 36%	OSCC 83% OSCC time diagnosis >60 yrs 30% Asymptomatic at early stage 27%	Tongue 74% Floor of the mouth 68% Tongue and floor of the mouth 46% Buccal mucosa 30% Palate 14%	Annual visual inspection for patients over 40 is mandatory 97.6% OSCC early diagnosis improves survival rate 96.4% Visual examination is effective in early detection 88% Up-to-date personal knowledge 83.7% Skills in neck examination 77.2% Lack of patients’ knowledge 67%	Tobacco 78.5% Tobacco products 65% Alcohol 40.5% Alcohol products 20%	Intra- and extraoral examination at 1st visit 33% Lymph nodes 33%
Yellowitz et al., 1995 [81]	57	>75%	N.A.	N.A.	OSCC time diagnosis >60 yrs 89% Pain 37.5%	N.A.	Annual visual inspection for patients over 40 is mandatory 92.5% Ease to referral 88.2% Up-to-date personal knowledge 73.1% OSCC early diagnosis improves the survival rate 65.5%	N.A.	N.A.

Abbreviations: OSCC: Oral squamous cell carcinoma; GDPs: General dental practitioners; OLP: Oral lichen planus; OSMF: Oral sub-mucous fibrosis; Max-fac surgeon: Oral and maxillofacial surgeon; ENT: Otolaryngologist; OM: Oral medicine; CE: Continuing education; RX: Radio diagnostics.

**Table 4:** Published data about medical practitioners’ KAP on OSCC.

			Knowledge				Attitude	Practice	
References	Participants	Quality Assessment	Risk Factors	Precancerous Lesions	Clinical Picture	Common Sites of Development	Opinion	History Taking	Physical Examination
Shanahan et al., 2018 [66]	221	>75%	Tobacco 93.7% OPMDs 69.1% Alcohol 63.3% HPV 29% Comorbidities 8.6% Betel quid chewing 5.9% Sun exposure 3.2% Diet 2.3%	Leukoplakia 34.5% Erythroplakia 14.5% Erythroleukoplakia 1.4%	Ulcer 67.3% Exophytes 31.4% Bleeding 15.5% Positive lymph node 3.2% Necrosis 1.4% Fixation 0.9% Induration 0.5%	N.A.	Visual examination is effective in early detection 14%	N.A.	N.A.
Shimpi et al., 2016 [67]	121	>75%	N.A.	Leukoplakia 65.3%	Abnormal growth 100% Ulcer 100%	N.A.	Smoking cessation is effective 100% Skills in neck examination 100% Ease for referral 56% Annual visual inspection for patient over 40 is mandatory 53% Undergraduate training was adequate 44%	N.A.	Intra- and extraoral 53%
Hassona et al., 2015 [74]	165	>75%	Tobacco 95.8% Prior OSCC 72.1% OPMDs 69.1% Comorbidities 57.6% Betel quid chewing 53.9% Alcohol 50.3% HPV 50.3% Elderly 47.9% Diet 31.5% Sun exposure 18.2%	Leukoplakia 61.2% OSMF 37.6% Candidiasis 37% Erythroplakia 35.2% OLP 27.9% Actinic cheilitis 13.9%	OSCC time diagnosis > 60 yrs 78.8% Dysphagia and limited tongue mobility 73.9% Positive lymph nodes 72.1% Lump 67.9% Ulcer 66.7% White patch 49.1% Non-healing socket 47.9% Red patch 44.2%	N.A.	N.A.	N.A.	Fluorescent imaging 77.6% Scalpel biopsy 73.3% Exfoliative cytology 56.4% Brush biopsy 43% Toluidine biopsy 17.6%
Hertrampf et al., 2014 [68]	192 GMPs 135 INTs 33 ENTs 28 DERMs	>75%	ENTS Tobacco 100%/Alcohol 100%/Prior OSCC 100%/Elderly 73% /HPV 70% /Sun exposure 64%/Diet 24% GMPs Tobacco 99%/Prior OSCC 94%/Alcohol 91%/Elderly 78%/HPV 54%/Sun exposure 46%/Diet 34% INTs: Tobacco 94.5%/Prior OSCC 92.5%/Alcohol 88.5%/Elderly 70.5% /HPV 50.5%/Sun exposure 48% /Diet 40% DERMs Sun exposure 96%/Prior OSCC 93%/Tobacco 93%/HPV 82%/Alcohol 79%/Elderly 75%/Diet 18%	ENTs Erythroplakia and leukoplakia 91% GMPs Erythroplakia and leukoplakia 85% INTs Erythroplakia and leukoplakia DERMs Erythroplakia and leukoplakia 82%	ENTs Positive lymph node 94% OSCC 91% OSCC diagnosis (III/IV stage) 85% Asymptomatic at early stage 27% GMPs OSCC diagnosis (III/IV stage) 85% Positive lymph node 82% OSCC 75% Asymptomatic at early stage 51% INTs OSCC diagnosis (III/IV stage) 85.5% Positive lymph node 85% OSCC 83.5% Asymptomatic at early stage 55% DERMs OSCC 93% OSCC diagnosis (III/IV stage) 82% Asymptomatic at early stage 82% Positive lymph node 79%	ENTs Floor of the mouth 67% Tongue 67% GMPs Floor of the mouth 71% Tongue 52% INTs Floor of the mouth 71% Tongue 60% DERMs Floor of the mouth 75% Tongue 61%	N.A.	N.A.	ENTs Intraoral 100% GMPs Intraoral 84% INTs Intraoral 78.5% DERMs Intraoral 89%
Tanriover et al., 2014 [69]	164	>75%	Tobacco 98.8% Prior OSCC 93.9% Poor oral hygiene 93.3% Alcohol 89% Family history 90.2% Elderly 87.8% Spicy foods 84.8% Sun exposure 73.2% Diet 68.9%	Erythroplakia and leukoplakia 84.1%	OSCC 75.6%	Floor of the mouth 51.8% Tongue 48.8 %	OSCC early diagnosis improves survival rate 87.8%	Tobacco 78.5% Tobacco products 70.1% Alcohol 56.7% Alcohol products 43.3%	Intra- and extraoral 65.2%
Alami et al., 2012 [75]	57	>75%	Tobacco 91% Alcohol 61% Elderly 48% Diet 25% HPV 22% Sun exposure 15%	Leukoplakia 64.9% Erythroplakia 17.5% OLP 12.3% Nicotinic stomatitis 12.3%	Positive lymph node 92.9% OSCC 89.3% Red or white patch 89.1% Asymptomatic at early stage 78.6% OSCC diagnosis (III/IV stage) 50%	Lips 42.1% Floor of the mouth 33.3% Tongue 22.8%	N.A.	N.A.	N.A.
Borhan–Mojabi et al., 2011 [76]	66	>75%	Tobacco 78.3% Alcohol 34.9%	N.A.	N.A.	Tongue 80.9% Lips 28.3% Floor of the mouth 25.7%	Adequate knowledge 51.5%	N.A.	Intraoral 39.4%
Reed et al., 2010 [77]	221	>75%	Betel quid chewing 98% Tobacco 90% Alcohol 37% HPV 18% Diet 4%	N.A.	N.A.	N.A.	N.A.	Alcohol 100% Prior OSCC 100% Tobacco 100% Alcohol products 97% Family history 97% Tobacco products 97%	N.A.
Applebaum et al., 2009 [78]	118	>75%	N.A.	Erythroplakia and leukoplakia 10%	N.A.	N.A.	Dentists are qualified to perform OSCC examination 91% Smoking cessation is effective 85% Alcohol cessation is effective 75% Physicians are qualified to perform OSCC examination 67% Visual examination is effective in early detection 46% Adequate knowledge 5%	Alcohol 100% Prior OSCC 100% Tobacco 100% Alcohol products 97% Family history 97% Tobacco products 97%	N.A.
Riordain et al., 2009 [70]	236	>75%	Tobacco 98.7% Alcohol 50.8% Poor oral hygiene 20.7% Elderly 5% Comorbidities 2.5% Ill-fitting prothesis 2.5% Spicy food 2.5% Dental caries 1.7% Male gender 1.2% Betel quid chewing 0.8% Gastric reflux 0.8%	Leukoplakia 12.7% OLP 1.2% Erythroplakia 0.4%	Ulcer 67.4% Pain 30.9% Swelling 21.6% Positive lymph node 16.5% Dysphagia 16.5% Bleeding 12.7% Lump 12.7% Halitosis 1.2% Hemoptysis 1.2% Burning sensation 0.8% Cough 0.8% Drooling 0.4% Hoarseness 0.4%	N.A.	Need of CE 99.6% Previous CE courses 3.39%	N.A.	N.A.
LeHew et al., 2009 [71]	8	<75%	Tobacco 87% Alcohol 62%	Leukoplakia 75% Erythroplakia 62%	N.A.	N.A.	N.A.	N.A.	N.A.
Nicotera et al., 2004 [72]	198	>75%	Tobacco 87.6% Alcohol 64% Prior OSCC 31.5% Elderly 2.8%	Leukoplakia 91.5% Erythroplakia 41.7%	OSCC 60.9% Red patch 17.6%	Tongue 68.8% Floor of the mouth 37.1%	Need of CE 84.9%	Tobacco 85.1% Alcohol 82.5% Prior OSCC 52.9% Family history 48.1%	Intra- and extraoral 63.8%
Macpherson et al., 2003 [79]	198	>75%	Tobacco 97% Alcohol 79% Elderly 76% HPV 23% Fungal infections 20%	Erythroplakia 22% Leukoplakia 72%	N.A.	N.A.	N.A.	N.A.	N.A.
Canto et al., 2002 [73]	240	>75%	Tobacco 100% Prior OSCC 99.2% Alcohol 89.3% Sun exposure 55.5% Elderly 42% Diet 29.5%	Erythroplakia and leukoplakia 10.4%	Positive lymph node 86.1% OSCC 80.2% Asymptomatic at early stage 71.3% OSCC diagnosis (III/IV stage) 60.1% Red patch 57% OSCC time diagnosis >60 yrs 42% Tongue high-risk area 34.5%	Tongue and floor of the mouth 25.4%	Skills in neck examination 98.8% Smoking cessation is effective 88.2% Alcohol cessation is effective 76% Visual examination is effective in early detection 61.8% Undergraduate training was adequate 53.8%	Alcohol 77% Alcohol products 77% Family history 77% Prior OSCC 77% Tobacco 77% Tobacco products 77%	Intraoral 90.7%
Greenwood et al., 2001 [80]	151	>75%	Tobacco 90.7% Betel quid chewing 50.3% Alcohol 45.7%	N.A.	N.A.	N.A.	N.A.	N.A.	Intra- and extraoral 68.2%
Yellowitz et al., 1995 [81]	93	<75%	N.A.	N.A.	OSCC time diagnosis > 60 yrs 92.6% Pain 50.7%	N.A.	Annual visual inspection for patients over 40 is mandatory 84.1% OSCC early diagnosis improves the survival rate 63.3% Ease for referral 51.3% Up-to-date personal knowledge 32.6%	N.A.	N.A.

Abbreviations: OSCC: Oral squamous cell carcinoma; ENT: Otolaryngologist; INTs: Intern; DERMs: Dermatologists; Max-fac surgeon: Oral and maxillofacial surgeon; OM: Oral medicine; OLP: Oral lichen planus; OSMF: Oral sub-mucous fibrosis; CE: Continuing education; OLP: Oral lichen planus; OSMF: Oral sub-mucous fibrosis; Max-fac surgeon: Oral and maxillofacial surgeon; OM: Oral medicine; CE: Continuing education.

## Data Availability

The data that support the findings of this study are available from the corresponding author upon reasonable request.
